# The Thaumarchaeon *N*. *gargensis* carries functional *bioABD* genes and has a promiscuous *E*. *coli ΔbioH*-complementing esterase EstN1

**DOI:** 10.1038/s41598-018-32059-0

**Published:** 2018-09-14

**Authors:** Jennifer Chow, Dominik Danso, Manuel Ferrer, Wolfgang R. Streit

**Affiliations:** 1 Microbiology and Biotechnology, University of Hamburg, 22609 Hamburg, Germany; 2 Institute of Catalysis, Consejo Superior de Investigaciones Científicas, 28049 Madrid, Spain

## Abstract

Biotin is an essential cofactor required for carboxylation and decarboxylation reactions in all domains of life. While biotin biosynthesis in most Bacteria and Eukarya is well studied, the complete pathway for this vitamer in Archaea is still not known. Detailed genome searches indicated the presence of possible *bio* gene clusters only in Methanococcales and Thaumarchaeota. Therefore, we analysed the functionality of the predicted genes *bioA*, *bioB*, *bioD* and *bioF* in the Thaumarchaeon *Nitrososphaera gargensis* Ga2.9 which are essential for the later steps of biotin synthesis. In complementation tests, the gene cluster-encoded *N*. *gargensis bioABD* genes except *bioF* restored growth of corresponding *E*. *coli* Rosetta-gami 2 (DE3) deletion mutants. To find out how biotin biosynthesis is initiated, we searched the genome for a possible *bioH* analogue encoding a pimeloyl-ACP-methylester carboxylesterase. The respective amino acid sequence of the ORF *estN1* showed weak conserved domain similarity to this class of enzymes (e-value 3.70e^−42^). Remarkably, EstN1 is a promiscuous carboxylesterase that complements *E*. *coli ΔbioH* and *Mesorhizobium loti ΔbioZ* mutants for growth on biotin-free minimal medium. Additional 3D-structural models support the hypothesis that EstN1 is a BioH analogue. Thus, this is the first report providing experimental evidence that Archaea carry functional *bio* genes.

## Introduction

Biotin-dependent carboxylases, decarboxylases and transcarboxylases are key enzymes of different ubiquitous reactions and are *e*.*g*. involved in CO_2_ fixation, fatty acid synthesis and amino acid catabolism^1,2^. With the exception of RuBisCO (ribulose-1,5-bisphosphate carboxylase/oxygenase), carboxylases from all domains of life require biotin as cofactor. Biotin can either be taken up from the environment or it can be synthesised as it has been shown for many bacteria, plants and fungi. Surprisingly, biosynthesis of biotin in Archaea has not been well studied.

**Figure 1:**
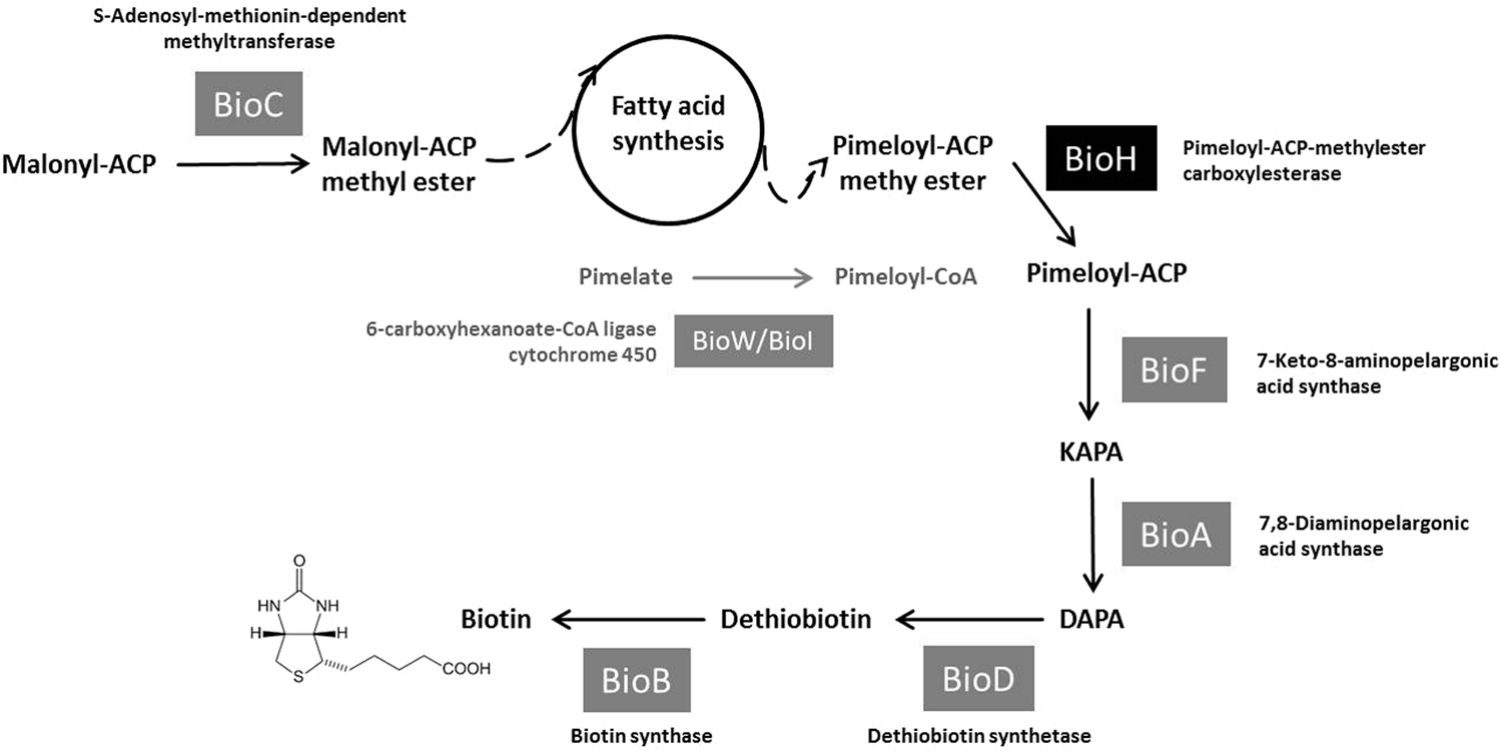
Synthesis pathway of biotin as it is known for Bacteria. In *E*. *coli*, BioC and BioH provide the pimeloyl moiety. In *Bacillus* spp., BioW produces the precursor molecule for biotin. The other genes *bioFADB* synthesizing the two fused rings and finally biotin are highly conserved^5^. *E*. *coli* BioF can convert both pimeloyl-CoA and -ACP while *Bacillus subtilis* BioF can only accept pimeloyl-CoA^42^.

Within Bacteria like *E*. *coli*, biotin is being synthesised with a *bioABCDF* operon and *bioH*. The first step of biotin synthesis, in which a biotin precursor is formed, is catalysed by BioC, a SAM-dependent methyl transferase (Fig. 1)^3^. The precursor molecule malonyl-ACP (acyl-carrier-protein) methyl ester is processed to pimeloyl-ACP methyl ester within the fatty acid synthesis cycle. This forms the substrate of BioH, a methyl ester carboxylesterase, which diverts pimeloyl-ACP methyl ester from the fatty acid cycle to the biotin synthesis pathway. BioH is thus responsible for a crucial step and has a “gatekeeper” function^4^. After hydrolysis and release of one molecule of methanol, pimeloyl-ACP undergoes an enzyme cascade finally leading to the biotin molecule^5^. In this process, a 7-keto-8-amino pelargonic acid (KAPA) synthase (BioF), a 7,8-diaminopelargonic acid (DAPA) aminotransferase (BioA), a dethiobiotin synthetase (BioD) and a biotin synthase [BioB, radical S-adenosylmethionin (SAM) superfamily] are involved. Regulation of this synthesis pathway is carried out by BirA, a biotin-protein ligase acting at the same time as transcriptional regulator of the *bio* gene cluster. Enzymes that carry out the same reaction like *E*. *coli* BioH and are thus also responsible for the cleavage of pimeloyl-ACP-methyl esters have been discovered for instance in *Haemophilus influenza* [BioG^6^], *Helicobacter pylori* [BioV^7^], *Francisella* species [BioJ^8^] and in cyanobacteria [BioK^9^]. The amino acid sequences of these enzymes differ clearly from each other and can be considered as evolutionary distinct although they have the same catalytic triad typical for α/β-hydrolases (usually formed by Ser, Asp/Glu and His) and similar structural properties^7,10^. In *Mesorhizobium loti*, BioZ shows amino acid sequence similarity to a β-ketoacyl-ACP synthase III, also known as FabH, but it was able to complement *E*. *coli ΔbioH* mutants^11^. While *bioH* in *E*. *coli* and *bioV* in *H*. *influenzae* are not integrated into the *bio* cluster, *bioH* in *P*. *aeruginosa* is and thus can be co-transcribed. The expression level of the operon-encoded *bioH* in *P*. *aeruginosa* is higher^12^. While the operon encoded genes *bioABDF* are highly conserved in Bacteria, *bioC* and *bioH* can be exchanged. In certain *Bacillus* species, the BioI (cytochrome P450) and BioW (pimeloyl-CoA synthetase) pathway has been discovered. Unlike the carbon-carbon bond cleaving BioI, BioW is essential for biotin synthesis and converts pimelic acid from the fatty acid synthesis pathway into pimeloyl-CoA^13^.

Compared to this variety of different enzymes involved in bacterial biotin synthesis, biotin synthesis in Archaea is far less investigated. By looking into genome datasets, it can be concluded that solely the euryarchaeal Methanococcales seem to have a complete set of biotin synthesis genes with *bioW*, *bioF*, *bioA*, *bioD* and *bioB*. Archaea from many other different clades like Lokiarchaeota, Korarchaeota, Sulfolobales and Thermococcales seem to contain only *bioB* genes. This leads to the assumption that these Archaea might synthesise biotin either in an unknown pathway or their biotin synthesis enzymes do not show enough similarity to their bacterial counterparts to be recognised as such. Other members of Thermoproteales, Methanosarcinales or Archaeoglobi might not be able to synthesise biotin at all as not even *bioB* can be found with database searches. Thaumarchaeota possess an incomplete set of biotin synthesis genes. Considering the impact and influential role they play for biogeochemical processes and for which they require biotin, their essential *bio* genes to fulfil these tasks have been investigated in this study more closely.

Thaumarchaeota represent the third largest archaeal phylum behind Euryarchaeota and Crenarchaeota^14^. Members of the thaumarchaeal lineage are highly abundant in marine, freshwater and soil ecosystems. Most of them are mesophilic ammonia-oxidisers that are capable of autotrophic CO_2_ fixation^15^. Additionally, some members are able to shift to a mixotrophic lifestyle by utilizing organic compounds^16^. The significant fixation rates of Thaumarchaeota for global carbon- and nitrogen-cycles have long been underestimated^17–19^. Still, thaumarchaeal genomes possess high numbers of genes with general function prediction only and even genes encoding hypothetical proteins with no known conserved domains at all. Within six thaumarchaeal genomes (*Nitrososphaera* spp. and *Nitrosocosmicus* spp.) deposited in the IMG/MER database^20^, between 46 and 51% of the predicted proteins are without function prediction, and therefore many pathways still remain incomplete.

In this study, we investigated the soil group I.1b Thaumarchaeon *Nitrososphaera gargensis* which was originally isolated from hot spring microbial mats and its hypothetical proteins involved in biosynthesis of the essential cofactor biotin. Genome analyses of the mesothermophilic ammonia-oxidiser *N*. *gargensis* revealed potential enzymatic pathways for the citric acid cycle, gluconeogenesis and the oxidative pentose-phosphate-pathway^21–23^. Like other ammonia-oxidizing Archaea (AOA), *N*. *gargensis* couples ammonia-oxidation with carbon fixation and assimilates inorganic carbon via an energy-efficient autotrophic 3-hydroxypropionate/4-hydroxybutyrate (HP/HB) cycle. This carbon-fixation pathway is even more efficient in Thaumarchaeota than in Crenarchaeota which use a modified HP/HB-pathway^17^. As the carboxylation reactions involved are biotin-dependent, they can be completely inhibited in presence of the specific inhibitor avidin. The *N*. *gargensis* genome is composed of 2.8 Mb of DNA encoding 3,562 genes of which so far only 52.4% have a function prediction (source: IMG). *N*. *gargensis* contains a predicted biotin synthesis gene cluster with *bioF*, *bioA*, *bioD* and *bioB*. Their function has not been demonstrated until now. The early stage synthesis genes *bioH*, *bioC* or *bioW/I* were not annotated or present as such. Our aim was to find out how this Archaeon produces biotin and therefore, the putative *bioFADB* genes and different α/β-hydrolases from *N*. *gargensis* that could have methyl ester carboxylesterase activity similar to BioH were studied for their functionality.

## Materials and Methods

### Cultivation of *N*. *gargensis* and genomic DNA extraction

*N*. *gargensis* was grown for at least eight weeks at 46 °C in a 300 ml Erlenmeyer flask containing 100 ml mineral salts medium^24^. For larger cell quantities, 5 l flasks containing 1.5 l of medium were incubated at 46 °C under slow stirring. The biotin-free medium was supplemented with trace element solution^25^ and contained 10 mM NaCl and 5 mM NH_4_Cl. The pH was controlled with cresol red indicator and was steadily adjusted with a solution of 5% (w/v) NaHCO_3_ to a pH of approx. 7.0. DNA was extracted from the cell pellet of a 1.5 l *N*. *gargensis* culture using a phenol-chloroform extraction method with RNase (1 mg/ml) containing lysis buffer and a proteinase K (10 mg/ml) incubation step for 1 h at 37 °C.

### Cloning of putative *bio*- and α/β-hydrolase-genes from *N*. *gargensis* into *E*. *coli* and activity screening on tributyrin (TBT)

From the genome sequence of *N*. *gargensis* (NCBI GenBank accession no. CP002408), the putative *bioF*, *bioA*, *bioD* and *bioB* genes were chosen for cloning (Table S1). Six different ORFs that were annotated as putative α/β-hydrolase fold proteins and could therefore serve as counterpart of the bacterial BioH were also selected as well as four possible candidates that could serve as *bioC* analogues (*bioC1-4*). The ORFs Ngar_c21820, Ngar_c24650, Ngar_c14400 (*i*. *e*. *estN1*), Ngar_c30910 (*i*. *e*. *estN2*), Ngar_c32780 and Ngar_c35080, which could possibly encode a BioH analogue, were amplified with specific primers, cloned into pDrive (PCR Cloning Kit, Qiagen, Hilden, Germany) and transformed into competent *E*. *coli* DH5α cells. As they encode carboxylesterases, the different clones were first tested on TBT agar plates at 37 °C for general activity. The plates were incubated for up to 6 days at 37 °C in order to see halos around active *E*. *coli* colonies expressing active carboxylesterases from *N*. *gargensis*. The genes *estN1* and *estN2* from the active clones and the *bioFADBC* genes were further separately cloned into the multiple-cloning site of pET21a(+) expression vectors (Novagen/Merck, Darmstadt, Germany) encoding a C-terminal six-fold histidine tag (His_6_-tag).

### Construction of biotin auxotrophic *E*. *coli* mutants

To test the functionality of the different *N*. *gargensis bio*-genes recombinantly, *E*. *coli* Rosetta-gami 2 (DE3) was chosen as a strain for creating insertion mutants of various biotin synthesis genes (*bioA*, *bioB*, *bioC*, *bioD*, *bioF* and *bioH*). Proteins from *N*. *gargensis* were much better expressed in this strain compared to *e*.*g*. BL21. Using the Gene Bridges “*E*. *coli* Gene Deletion Kit”, the manufacturer’s protocols were followed with prolonged incubation times as adjustment to the slower growth of Rosetta-gami 2 compared to other *E*. *coli* expression strains (approx. 1.5- to 2-times longer). As recombination plasmid, pRedET transferring ampicillin resistance was used. For each target ORF, a forward and reverse oligo nucleotide was designed that contained 50 bp of the target sequence and a homology arm region for the FRT-PGK-gb2-neo-FRT cassette that provides resistance to kanamycin (Table S1). Finally, the insertion mutants contained a 1,737 bp kan-cassette instead of their original gene. On medium without biotin, the strains were unable to grow.

### Functional complementation studies in *E*. *coli* on M9 medium

The *E*. *coli* Rosetta-gami 2 insertion mutants *ΔbioA*, *ΔbioB*, *ΔbioC*, *ΔbioD*, *ΔbioF* and *ΔbioH* were electroporated with the corresponding *E*. *coli* genes ligated into pET21a serving as controls to restore wild type activities and the corresponding genes from *N*. *gargensis* to check for their ability to complement growth (Table S1). Empty pET21a vector served as negative control. Syntheses of genes from *N*. *viennensis* EN76 (NCBI acc. no. AIC14436) and *N*. *evergladensis* SR1 (NTE_01571) with similarity to *estN1* were performed at Eurofins (Ebersberg, Germany). The corresponding genes were also cloned into pET21a and transformed into the *ΔbioH* mutants. All clones were tested for growth on M9 minimal medium^26^ with and without 4 nM D-biotin supplemented. The medium contained 0.8% (w/v) glucose, 100 µg/ml thiamin and 0.1% (w/v) casamino acids suitable for vitamin assays. As selection pressure and in order to induce expression of the plasmid-encoded genes under the T7 promoter, the agar plates contained 25 µg/ml chloramphenicol, 50 µg/ml ampicillin, 15 µg/ml kanamycin and 10 µg/ml IPTG. This IPTG amount showed the same result only with a faster colony growth compared to 100 µg/ml IPTG. After one to two days of incubation at 37 °C, the plates were checked for visible growth of colonies.

### Complementation of *Mesorhizobium loti ΔbioZ* on Rhizobial Defined Medium (RDM)

The genes *estN1* and *estN2* from *N*. *gargensis* as well as *bioH* from *E*. *coli* were cloned from pET21a including His-tag coding sequence into the broad-host-range vector pBBR1MCS-5 via the *Apa*I and *Pst*I restriction sites. *M*. *loti ΔbioZ*^11^ was transformed with the constructs and an empty vector control by triparental conjugation using *E*. *coli* DH5α as donor and *E*. *coli* K12 H101 with the helper plasmid pRK2013. *M*. *loti ΔbioZ* colonies were grown at 28 °C and selected on TY agar containing 200 µg/ml neomycin and 50 µg/ml gentamicin. In order to select for growth on biotin-free medium, RDM was prepared^27^ containing glucose (10 mM), trace elements^25^ and either none or 20 nM biotin.

### Overexpression of EstN1 in *E*. *coli* Rosetta-gami 2 (DE3) and enzyme purification

As mentioned above, the gene *estN1* (Ngar_c14400) encoding an active esterase was cloned into the pET21a expression vector (Table S1). The plasmid was transformed into *E*. *coli* BL21 [DE3; F^−^ *ompT hsdS*_g_(r_g_^−^m_g_^−^) *gal dcm*] and Rosetta-gami 2 [DE3; *Δara-leu7 679 ΔlacX74 ΔphoA PvuII phoR araD139 ahpC galE galK rpsL* F^−^(*lac lacI pro*) *gor522*::Tn10 *trxB* pRARE2] for comparative expression studies (both purchased from Novagen/Merck, Darmstadt, Germany). While the BL21 overnight culture containing *estN1*::pET21a was grown on medium with 100 µg/ml ampicillin, cultures of *E*. *coli* Rosetta-gami 2 strains were grown on LB medium supplemented with 34 µg/ml chloramphenicol, 3 µg/ml tetracycline and 100 µg/ml ampicillin. One litre of fresh LB medium containing the above mentioned antibiotics was inoculated in a 3 litre flask with 1% (v/v) of the overnight culture and incubated at 37 °C under stirring until an OD_600_ of 0.8 was reached. Therefore, the BL21 cultures were incubated for approx. 4 hours at 37 °C. The Rosetta-gami 2 cultures were slower in growth and reached an OD_600_ of 0.8 after approx. 7 hours. Overexpression of the enzyme was then induced by addition of IPTG to the cultures to a final concentration of 1 mM. The BL21 culture was incubated and stirred for further 4 hours at 37 °C and the Rosetta-gami 2 culture for further 16 to 18 hours at 28 °C. Cells containing recombinantly produced EstN1 were harvested by centrifugation, resuspended in lysis buffer and crude cell extracts were prepared by two passages through a French pressure cell press. Cell debris was collected by centrifugation. The target protein which contained a C-terminal His_6_-tag was purified from the clear supernatant via immobilised nickel ion affinity chromatography using Protino**®**Ni-NTA agarose (Macherey-Nagel, Düren, Germany) and eluted with a buffer containing 250 mM imidazole. The enzyme was dialysed and concentrated by centrifugation in a Vivaspin®6 ultrafiltration unit (MWCO 10,000; Sartorius, Goettingen, Germany) by washing twice with 0.1 M potassium phosphate buffer (pH 7.0). The protein was analysed by SDS-PAGE with gels containing 12% (separating gel) and 7% (stacking gel) acrylamide.

### Characterization of EstN1’s activity

#### Substrate spectrum on *para*-nitrophenyl esters (*p*NP)

Nine different *p*NP-ester compounds with a distinct residue chain length between 2 and 18 carbon atoms have been tested as substrates in a final concentration of 0.5 to 1 mM in 0.1 M potassium-phosphate buffer at pH 7.0. Unless otherwise indicated, this phosphate buffer was also used in the following assays. The reactions took place at 40 °C. 15 µg of purified EstN1 were added to 200 µl of the reaction mixture and incubated for 20 minutes before the reaction product *p*-nitrophenol was quantified spectrophotometrically at 405 nm.

#### Determination of esterase activity against *p*-nitrophenyl propionate (*p*NPP)

This substrate was tested in a final concentration of 1 mM in 0.1 M potassium-phosphate buffer at pH 7.0. The reaction was initiated by the addition of a stock solution (100 mM in acetonitrile) of *p*NPP to 195 µl of 0.1 M potassium-phosphate buffer containing 0.1 mg purified protein in 96-well microtiter plates. Reaction was followed at 405 nm by UV-VIS spectrophotometry in a Synergy HT Multi-Mode Microplate Reader. Initial rates were determined from linear fits of the absorbance versus time (<10% conversion) and corrected for the rate of un-catalysed hydrolysis. One unit (U) of enzyme activity was defined as the number of enzyme required to transform 1 µmol substrate in 1 min under the assay conditions using the reported extinction coefficient (∑_*p*NPP_ at 405 nm = 15200 M^−1^ cm^−1^). Reactions (performed in triplicate) were maintained at 40 °C.

#### Activity on mono- and dimethyl esters

Methyl acetate, methyl propionate, methyl butyrate, methyl hexanoate, methyl octanoate and dimethyl pimelate were tested as substrates. All substrates were diluted to a concentration of 100 mg/ml in dimethylsulfoxide (DMSO) and enzymatic hydrolysis was monitored in triplicates over 15 hours in a microtiter plate-based pH-shift assay using phenol red as indicator in a Synergy HT Microplate Reader at room temperature (25 °C). During hydrolysis, the increasingly acidic pH within the reactions was measured spectrophotometrically at 550 nm with an extinction coefficient of 8450 M^−1^ cm^−1^ ^28^.

#### Determination of acyl-CoA thioesterase activity

Acyl-CoA thioesterase activity was measured spectrophotometrically at 412 nm with 5,5′-dithiobis(2-nitrobenzoic acid) [DTNB^29^]. The medium contained 0.1 M potassium-phosphate buffer, 0.05 mM DTNB, 10 μM of acyl-CoAs (acetyl-CoA, n-propionyl-CoA, methylmalonyl-CoA, malonyl-CoA, succinyl-CoA or palmitoyl-CoA) and 0.1 mg purified protein. Reactions were followed at 412 nm by UV-VIS spectrophotometry in a Synergy HT Multi-Mode Microplate Reader. Initial rates were determined from linear fits of the absorbance versus time (<10% conversion) corrected for the rate of un-catalyzed hydrolysis. In all cases, one unit (U) of enzyme activity was defined as the number of enzyme required to transform 1 µmol substrate in 1 min under the assay conditions. An *E*_412_ = 13600 m^−1^ cm^−1^ was used to calculate the activity. Reactions were performed like in all other assays in triplicate and maintained at 40 °C.

#### Temperature optimum and temperature stability

Due to its higher stability against auto-hydrolysis, 0.5 mM *p*NP-C6 (hexanoate) was used for measuring the enzyme activity at different temperatures. To find the optimal temperature, the enzyme was pre-incubated between 10 °C and 90 °C for 5 minutes before the substrate was added to 0.5 mM concentration and the reactions were incubated for further 30 minutes. The temperature stability was tested by incubating the enzyme at 40 °C and 60 °C for up to 3.5 hours. The residual activity was then measured after addition of *p*NP-C6 and incubation at 40 °C for 30 minutes.

#### pH optimum

Enzyme reactions were carried out at 40 °C for 30 minutes with *p*NP-C6 using different citrate (pH 4.0 to 5.6), phosphate (pH 5.6 to 8.0), Tris-HCl (8.0 to 9.0) and glycine-NaOH (pH 9.0 to 10.6) buffers to find the optimal pH.

#### Influence of cofactors, solvent stability and stability against inhibitors and detergents

Ca^2+^, Co^2+^, Cu^2+^, Fe^3+^, Mg^2+^, Mn^2+^, Rb^2+^ and Zn^2+^ in 1 mM and 10 mM concentration were added to the standard enzyme reaction (30 min incubation at pH 7.0 and 40 °C with *p*NP-C6) in order to see if the enzyme activity depends on cofactors. As solvents, DMSO, isopropanol, methanol, dimethyl formamide (DMF), acetone, acetonitrile or ethanol were added to the standard reaction in 10% and 30% (v/v) concentration. As detergents, sodium dodecyl sulfate (SDS), Triton-X-100 and Tween80 [1 and 5% (v/v)] and as inhibitors in 1 and 10 mM concentration EDTA, dithiothreitol (DTT) or phenylmethylsulfonyl fluoride (PMSF) were tested to assay the residual activity after treatment with these substances.

### Database searches, structural prediction and Hidden-Markov-Model (HMM) search for similar enzymes

The genome sequence of *N*. *gargensis*^21^ was retrieved from the NCBI GenBank database [NCBI Resource Coordinators^30^] under accession no. CP002408. Together with other archaeal genomes deposited in the KEGG database (www.kegg.jp) and the Integrated Microbial Genomes (IMG) database of the Joint Genome Institute [https://img.jgi.doe.gov]^31^, the genomes were searched for genes related to the biotin biosynthetic pathway. Analysis of biotin synthesis enzymes from all archaeal phyla was accomplished using similarity analyses, KEGG pathway analyses, GenBank and IMG and included BioW, BioC, BioH, BioF, BioA, BioD and BioB. Conserved domain searches were performed using the NCBI web tool [https://www.ncbi.nlm.nih.gov/cdd]^32^ using amino acid sequences of the proteins of interest.

Amino acid sequences of two enzymes originating from closely related Thaumarchaeota that show relatively high similarity to EstN1 were retrieved from the NCBI database after performing BlastX and BlastP searches [NCBI Resource Coordinators]^30^. The sequences were aligned with T-coffee^33^ and a profile HMM was built with the alignment using the hmmbuild function of the HMMER package (http://hmmer.org). The Uniprot database^34^ was searched with this HMM to find similar sequences. These sequences were aligned and a new HMM was built by going through the same procedure as mentioned above. Highly conserved amino acid residues were identified after visual inspection of a HMM logo created with the webtool Skylign^35^. The relationship between the amino acid sequences of the different proteins was visualised in a neighbour-joining phylogenetic tree constructed with MEGA6^36^.

A homology model of the possible structure of EstN1 was calculated using the Robetta Full-chain Protein Structure Prediction Server [http://robetta.bakerlab.org]^37^ with a Ginzu prediction method. PDB files were created or retrieved from the PDB database (https://www.rcsb.org/) that was processed and visualised with the program Chimera [https://www.cgl.ucsf.edu/chimera]^38^. Pairwise comparison of protein structures was accomplished using the Dali server [http://ekhidna.biocenter.helsinki.fi/dali_lite]^39^.

## Results and Discussion

**Figure 2:**
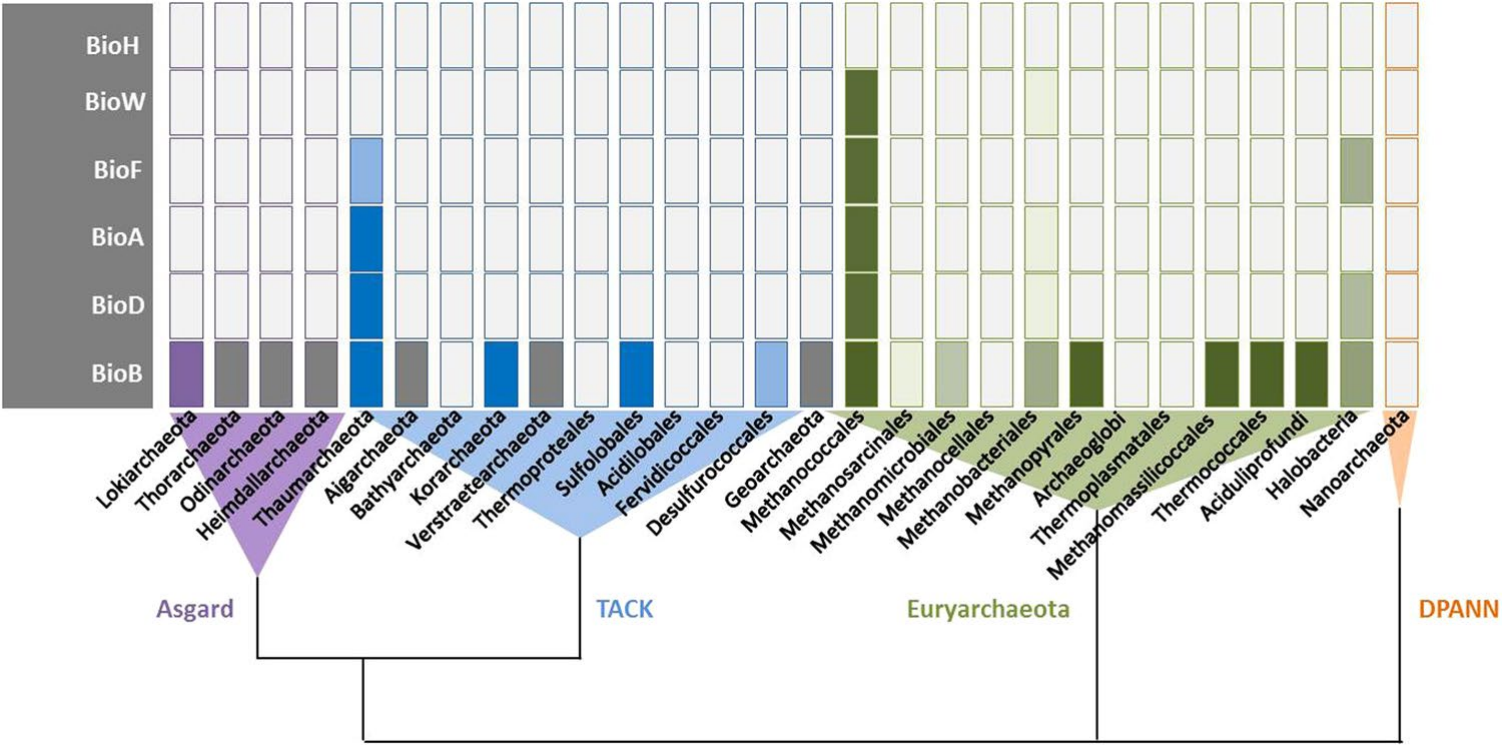
Biotin synthesis genes in Archaea. Presence of genes according to the KEGG and NCBI database. Colour shading indicates abundance within the different genera. Increase in colour intensity indicates higher abundance. Grey boxes: genomes not available in KEGG.

Within the KEGG database, 140 available archaeal genome sequences from 22 different phyla were searched for biotin synthesis genes (Table 1). In all but two phyla, the synthesis pathways are fragmentary although 119 of the 140 genomes contain a predicted *bioB* gene (Fig. 2). In Thaumarchaeota, genes for the synthesis starting from pimeloyl-CoA to biotin have been predicted. However no genes involved in the early synthesis steps were identified. The Methanococcales appear to be the only group within the archaeal domain that seems to have a complete biotin synthesis gene set and in which pimeloyl-CoA could be provided by the 6-carboxyhexanoate-CoA-ligase BioW. Remarkably, none of these genes has been verified for functionality.

**Table 1:** Putative biotin synthesis genes present in archaeal phyla according to the KEGG database.

Phylum (no. of species/strains)	*bioW*	*bioF*	*bioA*	*bioD*	*bioB*
Asgard group (1)					
• Lokiarchaeota (1)	0	0	0	0	2* (100%)
Euryarchaeota (140)					
• Methanococcales (18)	17 (94%)	17 (94%)	17 (94%)	17 (94%)	39* (100%)
• Methanosarcinales (29)	0	0	0	0	1 (3%)
• Methanomicrobiales (8)	0	0	0	0	3 (38%)
• Methanocellales (3)	0	0	0	0	0
• Methanobacteriales (13)	1 (8%)	0	1 (8%)	1 (8%)	8** (54%)
• Methanopyrales (1)	0	0	0	0	1 (100%)
• Archaeoglobi (8)	0	0	0	0	0
• Halobacteria (31)	0	18*** (52%)	0	14 (45%)	19 (61%)
• Thermoplasmatales (5)	0	0	0	0	0
• Methanomassilicoccales (2)	0	0	0	0	3** (100%)
• Thermococcales (20)	0	0	0	0	20 (100%)
• Aciduliprofundi [unclassified DHVE2 group (2)]	0	0	0	0	2 (100%)
Thaumarchaeota (12)					
(all except Caldiarchaeum have bioA/D/B)	0	0	11 (92%)	11 (92%)	11 (92%)
Crenarchaeota (57)					
• Desulfurococcales (14)	0	0	0	0	9 (64%)
• Sulfolobales (23)	0	0	0	0	25** (100%)
• Thermoproteales (17)	0	0	0	0	0
• Acidilobales (2)	0	0	0	0	0
• Fervidicoccales (1)	0	0	0	0	0
Cand. Korarchaeota (1)					
• *Cand*. Korarchaeum cyrptophilum	0	0	0	0	1 (100%)
*Cand*. Bathyarchaeota (1)					
• Bathyarchaeota archaeon BA1	0	0	0	0	0
Nanoarcheota (1)					
• *Nanoarchaeum equitans*	0	0	0	0	0

Genes encoding *bioC* and *bioH* are not known for Archaea in KEGG.

*All species contain a double set of *bioB* in the genome. **Some species contain two predicted *bioB* copies. ***Two species contain a double set of *bioF* in their genome.

Within this framework, we decided to investigate the functionality of the predicted *bio* genes in the phylum Thaumarchaeota and especially in *N*. *gargensis* as the way it obtains pimeloyl-ACP or -CoA biotin precursor molecules is not known.

### Complementation of *E*. *coli* Rosetta-gami 2 (DE3) *ΔbioF*, *ΔbioA*, *ΔbioD*, *ΔbioB* and *ΔbioC* mutants

**Figure 3:**
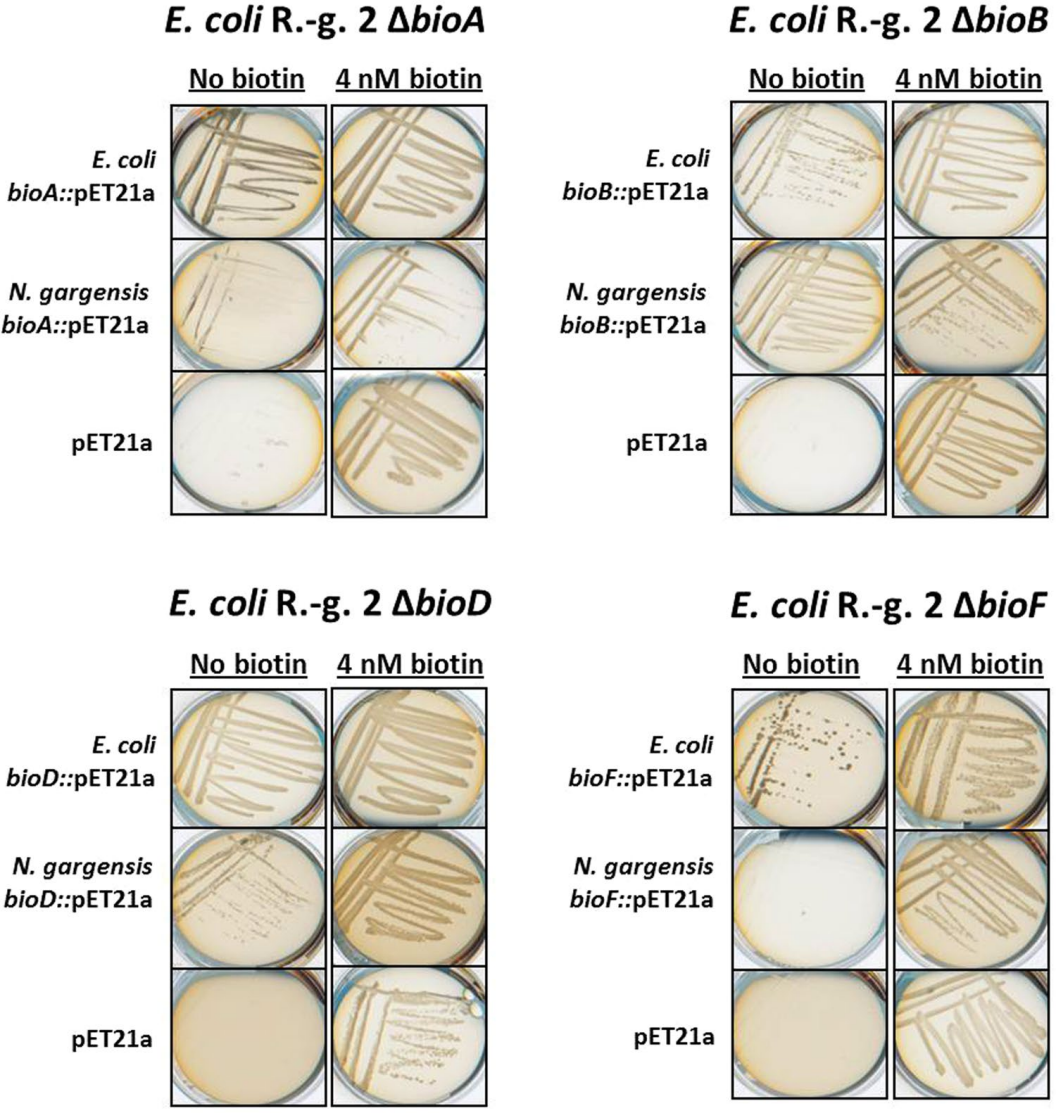
Auxotrophic insertion mutants of *E*. *coli* Rosetta-gami 2 (DE3) *bioA*, *bioB*, *bioD* and *bioF* were complemented with the corresponding *bioA*/*bioB*/*bioD*/*bioF*-genes from *E*. *coli* and *N*. *gargensis* and an empty vector pET21a served as control. The clones were assayed for growth on M9 medium without biotin and under supplementation of 4 nM biotin. All complementation assays were carried out in triplicate in at least three independent experiments.

Because of the large phylogenetic distance of the *N*. *gargensis* genes to *E*. *coli* and the known problems concerning expression of archaeal proteins, *E*. *coli* Rosetta-gami 2 was chosen as host for complementation assays. It carries genes for seven additional tRNAs that help to solve the rare codon usage problem and increase protein yield^40^ as well as *trx*B/*gor* mutations for enhanced disulfide bond formation^41^. To investigate the functionality and compatibility of *N*. *gargensis* genes annotated as enzymes involved in biotin synthesis, five different auxotrophic *E*. *coli* Rosetta-gami 2 mutant strains were created. Each of the mutants *ΔbioF*, *ΔbioA*, *ΔbioD*, *ΔbioB* and *ΔbioC* carries a kanamycin resistance gene instead of the respective gene. The mutants were verified by PCR and failed to grow on M9 media not supplemented with nanomolar amounts of biotin. Furthermore, the mutant strains transformed with an empty pET21a vector as negative control also failed to grow without biotin (Fig. 3). In our experiments, the mutant strains were complemented with the corresponding *E*. *coli* gene and with the putative *N*. *gargensis* counterpart. On biotin-free medium, *E*. *coli ΔbioA*, *ΔbioB* and *ΔbioD* showed sufficient growth when complemented with both the *E*. *coli* and the *N*. *gargensis* version of the corresponding genes. *E*. *coli ΔbioA* and *ΔbioD* could be restored to approximately half of the full growth by *N*. *gargensis*’ *bioA* and *bioD*. The *E*. *coli ΔbioF* mutant, however, could only be complemented with *E*. *coli*’s *bioF* and not with the *N*. *gargensis* equivalent (Fig. 3). Since *bioDABF* are located in a gene cluster (NCBI acc. no. Ngar_c17540-Ngar_c17580), we speculate that *bioF* is active, but maybe only on pimeloyl-CoA and not on pimeloyl-ACP provided by *E*. *coli*. This is also the case for *Bacillus subtilis* BioF^42^ and would make sense as no ACPs are known for Archaea^43^.

An *O*-methyltransferase and *bioC*-analogue was not found in the genome. Concerning *bioC*, only four out of 15 possible *bioC* candidates annotated as methyltransferases from *N*. *gargensis* were tested and failed to complement the *E*. *coli ΔbioC* mutant. Further research is necessary in this case.

We conclude that *N*. *gargensis* possesses at least the functional biotin synthesis genes *bioA*, *bioD* and *bioB*.

### Conserved domains of the six putative α/β-hydrolases from *N*. *gargensis*

Six different α/β-hydrolase genes without further specified function prediction were chosen from the *N*. *gargensis* genome in order to be tested for functionality and for possible BioH-activities. According to a conserved domain search, three of the candidate enzymes (Ngar_c21820, Ngar_c24650 and Ngar_c35080) showed domains for being members of the α/β-hydrolase superfamily (e-values between 1.9e^−44^ and 2.1e^−17^), while three others even showed possible pimeloyl-ACP methyl ester carboxylesterase domains [Ngar_c14400 (*i*.*e*. EstN1), Ngar_c30910 (*i*.*e*. EstN2) and Ngar_c32780; e-values between 4.2e^−41^ and 2.0e^−20^].

### Hydrolytic activity of carboxylesterases from *N*. *gargensis* after expression in *E*. *coli*

The six different putative α/β-hydrolase genes Ngar_c21820, Ngar_c24650, Ngar_c14400 (*estN1*), Ngar_c30910 (*estN2*), Ngar_c32780 and Ngar_c35080 were cloned into *E*. *coli* DH5α. As it is known that BioH enzymes are slightly promiscuous and able to hydrolyse the triglyceride tributyrin (TBT^44^), the respective clones were screened for activity on LB agar plates containing 1% of TBT. This step was performed to ensure that only active enzymes were further pursued. After incubation of one day at 37 °C and one day at 46 °C, three of the clones showed reproducible activity and can therefore be further specified as carboxylesterases (EC 3.1.1). The clones contained the genes *estN1* (786 bp), *estN2* (834 bp) and Ngar_c35080 (789 bp). Due to its very low activity, the clone containing Ngar_c35080 was not further investigated.

### Sequence analysis of the *N*. *gargensis* carboxylesterases EstN1 and EstN2

The amino acid (aa) sequences of the two enzymes EstN1 (261 aa, estimated size of 29.6 kDa) and EstN2 (277 aa, estimated size of 31.1 kDa) show similarities to enzymes from other Thaumarchaeota. EstN1 (GenBank AFU58376) has similarity to other not further specified putative α/β-hydrolases from a soil-metagenome-derived Thaumarchaeon (89% identity, e-value 1e^−175^), *Candidatus* Nitrososphaera evergladensis SR1 (72%, 8e^−132^) and two *Nitrososphaera viennensis* strains (72%, 8e^−132^ and 1e^−131^, respectively) according to a BlastP search. It has an identity of less than 52% to other thaumarchaeal hydrolases. In case of EstN2 (GenBank and PDB acc. no. 5A62_A^45^), only one enzyme from *Nitrososphaera evergladensis* SR1 annotated as putative hydrolase or acyltransferase of the α/β-superfamily shows a fairly higher similarity with 70% identity and an e-value of 1e^−130^. The next best hits belonged to *Sulfolobus islandicus* strains (30–31% identity, e-values 4e^−25^ to 2e^−24^), but also to Bacteria like *Chloroflexi* (29%, 5e^−28^) or *Rhodobacter* sp. (30%, 2e^−26^).

### Complementation studies in *E*. *coli* Rosetta-gami 2 *ΔbioH* and *Mesorhizobium loti ΔbioZ* mutants

**Figure 4:**
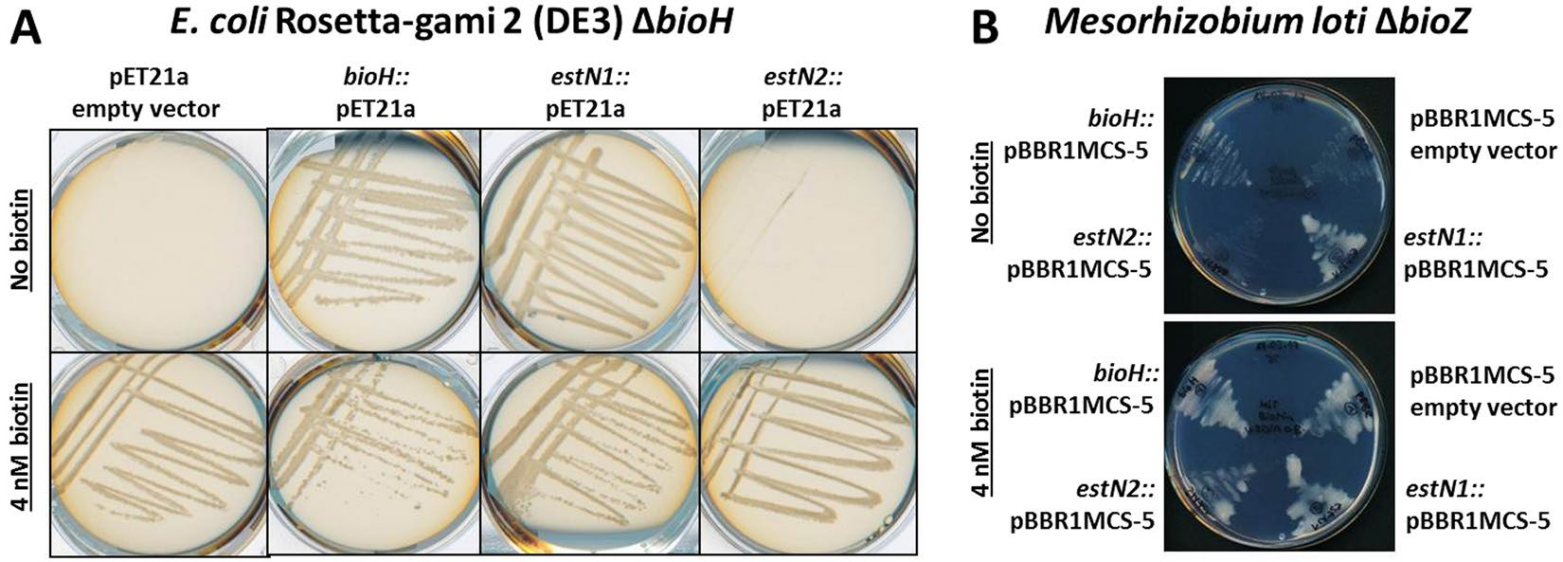
Complementation of *E*. *coli ΔbioH* (**A**) and *M*. *loti ΔbioZ* (**B**) auxotrophic mutants on M9 and RDM medium. The mutants were complemented with empty vector and the plasmid encoded genes from *E*. *coli* and *N*. *gargensis*. The *N*. *gargensis* gene *estN1* was able to restore growth in both bacterial strains in contrast to the other esterase *estN2*.

BioH is an essential enzyme for biotin biosynthesis^46^ and *ΔbioH* mutants are dependent on biotin supplementation. An *E*. *coli* Rosetta-gami 2 *ΔbioH* mutant was created and transformed with pET21a as an empty vector control, with *bioH*::pET21a from *E*. *coli* to restore wild-type growth, and with *estN1* and *estN2* ligated into pET21a. After one to two days of incubation at 37 °C, all strains were grown on M9 medium with 4 nM biotin supplemented. On biotin-free medium, only the mutant strains containing *bioH*::pET21a and *estN1*::pET21a were able to grow (Fig. 4A). Thus (unlike EstN2) EstN1 catalyses the same hydrolysis reaction on pimeloyl-ACP-methyl ester like BioH.

Within the α-proteobacterium *M*. *loti*, the 3-oxoacyl-ACP-synthase BioZ bypasses BioH and leads to pimeloyl-ACP production in another way. BioZ is a FabH (β-ketoacyl-ACP synthase) homologue and not an α/β-hydrolase. It has a catalytic Cys/His/Asn triad^47,48^. Thus, it is not able to cleave the methyl ester like BioH with its catalytic Ser. However, *E*. *coli*’s BioH complemented an auxotrophic *M*. *loti ΔbioZ* mutant^11^ and therefore, *M*. *loti ΔbioZ* was transformed with an empty broad-host-range vector pBBR1MCS-5 as well as with *bioH*, *estN1* and *estN2* in pBBR1MCS-5. All strains were able to grow at 28 °C on RDM medium supplemented with 20 nM biotin after four days. Without the addition of biotin, growth was significantly impaired for cells containing the empty vector and *estN2*, while *E*. *coli*’s *bioH* was able to restore wild-type growth which is consistent with previous findings^11^. Growth of *M*. *loti ΔbioZ* was even stronger with *estN1* that could apparently fully complement *bioZ* (Fig. 4B).

### Recombinant production and biochemical characterization of EstN1

EstN1 was overexpressed with a C-terminal His_6_-tag in *E*. *coli* BL21 and Rosetta-gami 2 to study its enzymatic properties. As previously mentioned, Rosetta-gami 2 contains an extra plasmid providing tRNAs for rare codons and imparts improved disulfide bond formation and protein folding. The enzyme was expressed in this strain with much higher yields compared to BL21. The enzyme was purified with Ni-NTA-agarose (Fig. S1) and subjected to buffer change and concentration before its activity was characterised. EstN1 was mostly active on short-chain *p*NP-esters as model substrates. On *p*NP-esters, the highest esterase activity was measured with 10.1 ± 0.19 U/mg on *p*NPP (-C3). It was significantly higher than the activity observed for other shorter or longer *p*NP esters, namely, *p*NP-acetate (-C2) with 0.2 U/mg followed by 0.1 U/mg on *p*NP- butyrate (- C4) and 0.07 U/mg on *p*NP-hexanoate (-C6). This substrate spectrum on *p*NP esters and the preference for short acyl chains are very similar to the ones of *E*. *coli* BioH^49^.

The carboxylesterase was mostly active (>60% relative activity) in a moderately thermophilic range between 30 and 55 °C in agreement with the optimal growth temperature of *N*. *gargensis* (∼46 °C). The highest activity was measured at 40 °C. Beneath 20 °C and above 60 °C, only low activity could be detected (<35% rel. activity) (Fig. S2, left). EstN1 is most active in a range between pH 5 and 8 with an optimum at pH 6 in phosphate buffer (Fig. S2, middle). This differs to the rather narrow pH optimum of BioH from *E*. *coli* (pH 8.0–8.5). EstN1 has a half-life-time (50% residual activity) of approx. 135 minutes at 40 °C and 15 minutes at 60 °C (Fig. S2, right).

EstN1 showed activity on methyl esters, but the activity was comparably low with 0.06 U/mg on methyl hexanoate. The activity was lower on shorter and longer residues. A higher activity was observed for dimethyl pimelate with 0.35 U/mg. Dimethyl pimelate resembles the proposed natural substrate pimeloyl-ACP-methyl ester; still, it is a much smaller molecule. Concerning the specific activity, it is noteworthy that the monomethyl and dimethyl ester assays could not be carried out at 40 °C due to restraints in experimental setup, but at room temperature (25 °C) and that the values are expected to be higher under optimal conditions.

EstN1 was finally tested for thioesterase activity against diverse acyl-CoAs to learn more about its promiscuity and possible activity on substrates similar to pimeloyl-CoA. It showed its overall highest activity at 40 °C towards n-propionyl-CoA (14.6 ± 0.3 U/mg) and to a much lower extent for acetyl-CoA (0.26 ± 0.03 U/mg), but no considerable activity on any of the other derivatives tested with more complex residues. Given the fact that thirteen out of 22 structurally different substrates were hydrolysed, EstN1 can be considered as enzyme with slightly promiscuous activity.

EstN1 is a cofactor-independent carboxylesterase as none of the tested ions Ca^2+^, Co^2+^, Cu^2+^, Fe^3+^, Mg^2+^, Mn^2+^, Rb^2+^ and Zn^2+^ enhanced its activity significantly. It was susceptible to enzyme inhibitors and retained between 30% (10 mM PMSF) and 66% (10 mM EDTA) residual activity after treatment for 15 minutes. DTT diminished its activity completely. While Triton X-100 and Tween 80 did not significantly affect the enzyme, SDS inhibited EstN1 completely. When treated with the solvents DMSO, isopropanol, methanol, DMF, acetone, acetonitrile and ethanol, EstN1 generally kept its activity between 81% (isopropanol) or even enhanced it to 105% (DMF) when 10% of the solvents were applied. In higher concentrations of 30%, the solvents decreased the enzymatic activity to values between 43% (acetone and isopropanol) and 90% (acetonitrile). DMSO and DMF enhanced the activity in 30% concentration to 123 and 108%, respectively.

### Homology model of EstN1

**Figure 5:**
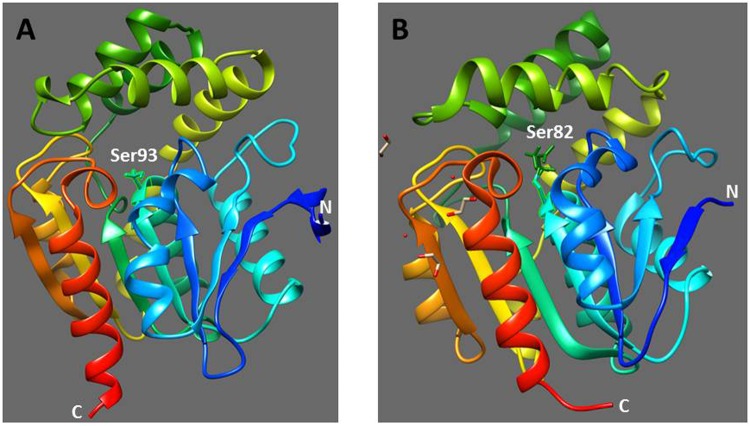
Structural comparison between the homology modelled enzyme structure of *N*. *gargensis* EstN1 (**A**) and the crystal structure of *E*. *coli* BioH (**B**); (PDB 1M33). The catalytic serine residue of both structures as well as the N- and C-termini are indicated. The EstN1 structure was calculated with Robetta using the Ginzu prediction protocol.

A model of EstN1’s protein structure has been calculated with the prediction server Robetta and the Ginzu PDB template identification and domain prediction protocol. Regions of EstN1’s protein chains were aligned to PDB templates. Altogether, the structural model has been calculated out of 15 different X-ray structures of α/β-hydrolases from different Bacteria like *Burkholderia xenovorans* (PDB code 2XUA), *Pseudomonas fluorescens* (2D0D and 1IUN) and a metagenomic α/β-hydrolase (4Q3L). The identities were rather low with a maximum of 29.45% to a meta-cleavage product hydrolase CumD from *P*. *fluorescens* (2D0D). The comparative model shows a central β-sheet composed of eight parallel β-strands, one of them in antiparallel orientation (Fig. 5A). The catalytic Ser93, His238 and Asp211 are positioned in the centre and can be accessed by small molecules through a hydrophobic tunnel (Fig. S3A). Intriguingly, the protein structure of BioH from *E*. *coli* (1M33) was not considered in this automated modelling process, because its amino acid sequence similarity to EstN1 is too low (22% on the total sequence). As both enzymes catalyse the same reaction, the structure of BioH was retrieved from the PDB database and compared to EstN1 manually (Fig. 5B). For BioH, a proof that pimeloyl-ACP-methyl ester is the natural substrate has been made by co-crystallization with this substrate^4^. The structures of EstN1 and BioH show many similarities regarding their tertiary structure, surface hydrophobicity and architecture of their catalytic domain (Fig. S3). The structural similarity between the two enzymes is given with a Z-score of 28.8 and a RMSD (root-mean-square deviation) of 2.3 based on a pairwise structural alignment using the DALI tool. It is visualised by superimposition of the two structures (Fig. S3C). Next to its highly similar tertiary structure, BioH has a catalytic triad composed of a Ser82, His235, and Asp207^49^, but the catalytic serine is surrounded by a GWSLGG motif in comparison to the GSSFGG motif of the three enzymes from the *Nitrososphaera* species which have been taken into account in a sequence alignment (Fig. S4). It is known that esterases like BioG from *Haemophilus influenza*, BioK from cyanobacteria, BioJ from *Francisella* spp. and BioV from *Helicobacter* spp. have the same function like BioH^7^. The enzymes can be structurally different from each other, but, based on homology models, all have similar components including eight β-sheets, six α-helices and the catalytic residues Ser, His and Asp situated on loops.

### Comparison between EstN1 and similar esterases from *N*. *evergladensis* and *N*. *viennensis*

**Figure 6:**
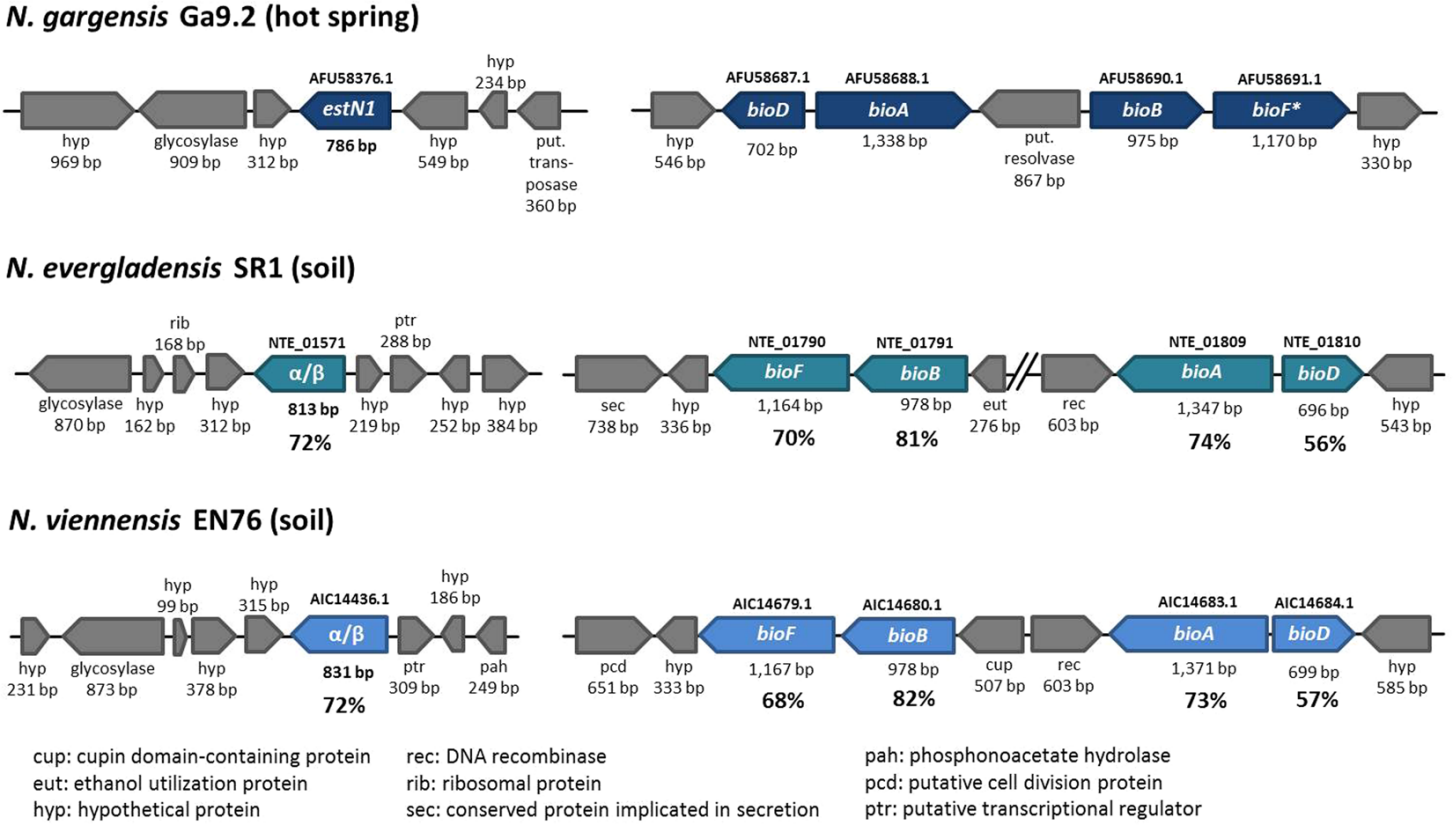
Biotin synthesis operons of three *Nitrososphaera* species. ORF context of *estN1* and genes from *N*. *evergladensis* and *N*. *viennensis* with the highest similarity to *estN1*. Function prediction for *N*. *gargensis* and *N*. *evergladensis* retrieved from NCBI, for *N*. *viennensis* from IMG (coding regions named according to the NCBI database). The amino acid sequence identities of the *N*. *evergladensis* and *N*. *viennensis* biotin synthesis proteins to the amino acid sequences from *N*. *gargensis* are indicated as percentages beneath the corresponding genes. *Functional proof is missing.

Next to an enzyme from an uncultured Thaumarchaeon (OLC87659), the two esterases from the closely related *N*. *evergladensis* SR1 (AIF83634) and *N*. *viennensis* EN76 (WP_084790873) show the highest similarity to EstN1 on amino acid level with an identity of 72% and an E-value of 8e^−132^. The two esterases were synthesised, cloned into *E*. *coli* Rosetta-gami 2 *ΔbioH* and tested for their ability to perform the same BioH activity as EstN1. However, the enzymes were not able to complement for growth on M9 agar plates without biotin despite their relatively high similarity to EstN1 (Fig. S5). Within the amino acid alignment of the three sequences together with BioH, some residues become apparent that only EstN1 and BioH have in common and might explain this difference (Fig. S4). Only few amino acid substitutions can shift the activity of a carboxylesterase towards BioH activity^50^. It was supposed in previous studies that an esterase which is actually involved in another pathway could have shifted its activity by successive point-mutations and gene duplications to a higher specificity for pimeloyl-ACP-methyl esters^7,51^. This protein evolution could have happened in *N*. *gargensis* and could also explain EstN1’s slightly promiscuous activity on other substrates than pimeloyl-ACP or -CoA methyl ester. The biotin synthesis operon of *N*. *evergladensis* and *N*. *viennensis* are arranged differently to the operon from *N*. *gargensis* which is also a sign that the ability to synthesise biotin evolved separately within the species (Fig. 6). It is possible that within these two *Nitrososphaera* species another esterase has taken over the pimeloyl-ACP or -CoA methyl esterase function or perhaps only the CoA-form can be hydrolysed. Nevertheless, it cannot be fully excluded that EstN1 rather coincidentally cleaves pimeloyl-ACP methyl ester and randomly fulfils the BioH activity with its promiscuous activity on methyl esters, an activity that the other two enzymes might not have in the same way.

### Phylogenetic analysis of EstN1 homologues based on amino acid sequence similarities

**Figure 7:**
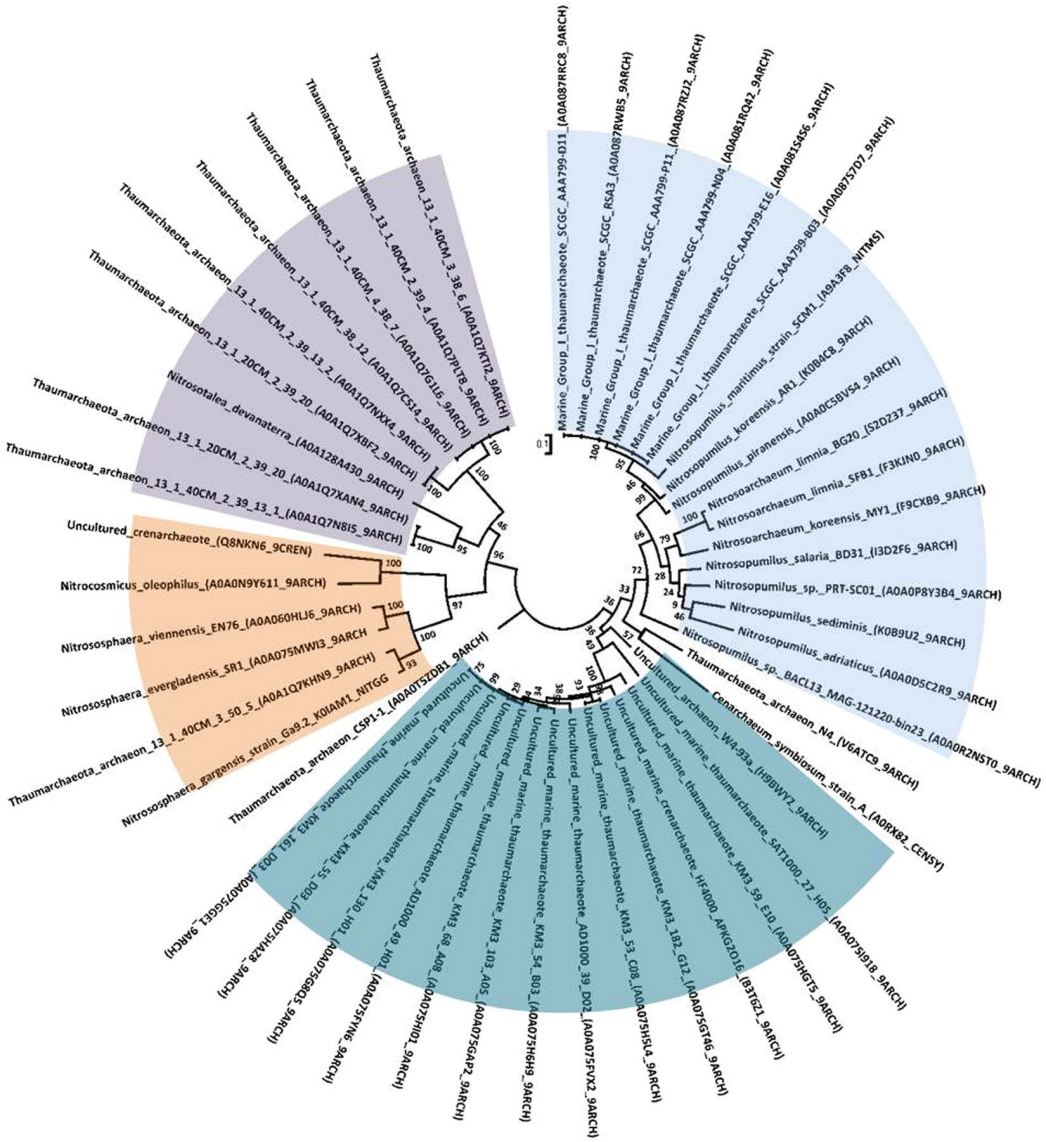
Phylogenetic reconstruction of protein sequences found with our HMM-based search within the Uniprot database using the Maximum Likelihood Method. Search for similar amino acid sequences was carried out using a HMM model constructed out of five different sequences with high similarity to EstN1 and resulted in 49 different thaumarchaeal sequences. Number of Bootstrap Replications: 100. Uniprot accession numbers are indicated in brackets. The tree can be grouped into at least four different branches. Orange: *Nitrososphaera* lineage; purple: *Nitrosotalea* lineage; light blue: *Nitrosopumilus* lineage; petrol: marine uncultured lineage.

We took amino acid sequences of the α/β-hydrolases from *N*. *evergladensis* SR1 and *N*. *viennensis* EN76 that showed the highest similarity to EstN1 in order to construct a Hidden-Markov-model (HMM) that points out conserved residues and motifs. Application of this HMM in Uniprot database searches with a significance E-value of 0.01 set as cut-off resulted in two other Thaumarchaeal α/β-hydrolase hits from a metagenomic Thaumarchaeon 13_1_40cm (GenBank OLC87659) and *Candidatus* Nitrosotenuis chungbukensis (WP_042686824). A refined HMM was calculated out of these five sequences and applied for a new database search that resulted in 49 hits. All hits belonged to members of the Thaumarchaeota and a large fraction originates from uncultivated marine Thaumarchaeota (Fig. 7). Other hits group to members of the genus *Nitrosopumilus*, *Nitrosotalea* or *Nitrososphaera*. The HMM logo (Fig. S6) leading to these sequences shows the conserved catalytic triad with a Ser (position 96), Asp (pos. 214) and His (pos. 242). Highly conserved amino acids are furthermore tryptophan (W, pos. 38, 210), proline (P, pos. 43, 65,142, 176, 218, 244, 249) and glycine (G, pos. 30, 31, 58, 60, 123, 211). Three other glycine residues (pos. 94, 98, 99) surround the serine within the catalytic triad and form a GSSXGG motif.

### Synthesis of biotin in Archaea

Many metabolic processes are strictly dependent on biotin. Within 14 out of 21 different phyla, at least a few representatives contain a biotin synthase *bioB* gene and therefore should be able to synthesise the cofactor (Fig. 2; Table 1). Considering the extreme environmental conditions in many archaeal habitats, biotin uptake into the cells of extremophiles is often impossible. Biotin is generally sensitive to both acids and bases, but relatively stable against light, heat or oxidizing and reducing agents (source: https://toxnet.nlm.nih.gov/). Mostly mesophilic and anaerobic Methanosarcinales, however, possess biotin-dependent enzymes like pyruvate carboxylases but do not have biotin synthesis genes. They have a BioY biotin uptake transporter instead.

Three different classes of enzymes require biotin as cofactor to translocate carbon dioxide, namely carboxylases, decarboxylases and transcarboxylases. Carboxylases catalyse CO_2_-fixation and enzymes like pyruvate carboxylase (gluconeogenesis), acetyl-CoA carboxylase (fatty acid synthesis), propionyl-CoA carboxylase (amino acid and fatty acid oxidation), beta-methylcrotonyl-CoA carboxylase (amino acid catabolism), geranyl-CoA carboxylase (isoprenoid catabolism) and urea amidolyase (nitrogen metabolism) belong to this group^2^. Acetyl-CoA carboxylase and propionyl-CoA carboxylase are the CO_2_-fixing enzymes in the 3-hydroxypropionate bicycle and the 3-hydroxypropionate-4-hydroxybutyrate cycle and are therefore mandatory for Archaea with these autotrophic carbon fixation pathways^52^. Depending on their metabolism, most members of the Archaea are able to perform at least one pathway that requires biotin. Exceptions are for instance the obligate symbiotic *Nanoarchaeum equitans* (Nanoarchaeota) or Thermoplasmatales that do not seem to have biotin dependent carboxylases. One possible BioB that occurs in the KEGG database from a Thermoplasmatales archaeon might as well be a radical SAM protein according to a BlastX search. How biotin synthesis *e*. *g*. in Sulfolobales and Thermococcales takes place remains to be elucidated as no enzymes with similarity to the other biotin synthesis genes could be found (Table 1). While Methanococcales possess the genetic equipment for a *Bacillus*-like *bioW* pathway, this study shows that Thaumarchaeota like *N*. *gargensis* could be able to synthesise biotin following the *E*. *coli*-like *bioH* pathway.

Still, many open questions remain about be origin of the pimeloyl-CoA methyl ester required for biotin synthesis as until now, no fatty acid synthesis pathway for Archaea is known although several Archaea have been reported to be capable of performing it^53^. A possible fatty acid biosynthesis pathway that is currently being discussed for Archaea could function like the reversed β-oxidation of fatty acids^53^. It could similarly work with CoA instead of ACP. The enzymatic equipment proposed for this pathway is present in *N*. *gargensis* as it possesses an acetyl-CoA C-acetyltransferase from the mevalonate pathway (NCBI-acc. no. Ngar_c00180) that catalyses the transfer of C_2_ fragments on acetyl-CoA. The proposed fatty acid synthesis could continue with a 3-hydroxyacyl-CoA dehydrogenase (Ngar_c14000), which is also annotated as enoyl-CoA hydratase, producing the intermediate acyl-2-enoyl-CoA. Finally, an acyl-CoA dehydrogenase (Ngar_c13350) and a possible ketoacyl-CoA thiolase (Ngar_c17490) could complete this possible biosynthesis pathway. *N*. *gargensis* has two putative adjacent acetyl-CoA/propionyl-CoA carboxylases (Ngar_c01870 and Ngar_c01880) that could convert acetyl-CoA into malonyl-CoA for the initiation of biotin synthesis. According to KEGG, *N*. *gargensis* might even encode a solitary 3-ketoacyl-ACP-reductase FabG (Ngar_c11430). Experimental verification of this hypothesis is needed.

## Conclusions

Our study shows that *bioA*, *bioB* and *bioD* from *N*. *gargensis* are functional and able to complement *E*. *coli* deletion mutants. This is a first function-based proof of biotin synthesis in Thaumarchaeota and in the Archaea in general. Furthermore, the investigated and characterised carboxylesterase EstN1 has a full BioH activity and striking structural similarities to its bacterial counterpart although no significant similarity can be found on sequence-level. BioH is the key-enzyme that takes precursor molecules from the fatty acid cycle in order to feed them into the biotin-forming enzyme cascade. The significance of this study lies in the fact that essential global carbon cycles Archaea are involved in and metabolic pathways they possess strictly depend on biotin as cofactor.

## Electronic supplementary material

Supplementary Information
