# Supplementary material for: The Thaumarchaeon N. gargensis carries functional bioABD genes and has a promiscuous E. coli ΔbioH-complementing esterase EstN1
Source: Sci Rep. 2018 Sep 14;8:13823. doi: 10.1038/s41598-018-32059-0 (PMC6138646; doi:10.1038/s41598-018-32059-0)
Supplement: Supplementary file 1 — Supplementary Information [file 41598_2018_32059_MOESM1_ESM.pdf]

Supplementary Information

The Thaumarchaeon *N. gargensis* carries functional *bioABD* genes and has a promiscuous *E. coli ΔbioH*-complementing esterase EstN1

Jennifer Chow[1], Dominik Danso[1], Manuel Ferrer[2] and Wolfgang R. Streit[1] *

[1] Microbiology and Biotechnology, University of Hamburg, 22609 Hamburg, Germany

[2] Institute of Catalysis, Consejo Superior de Investigaciones Científicas, 28049 Madrid, Spain

*Corresponding author.

Table S1: List of primers used in this study. Restriction sites are underlined.

Target and primer designation	Forward primer sequence (5´→3´)	Reverse primer sequence (5´→3´)
Ngar_c21820_for / _rev	GGGAATCCAGCACGAAGATTAG	CATCAAGACGAGAAATCACGAATC
Ngar_c24650_for / _rev	ACAGCGCTTCATATCATACTACC	ATAGCTGTCTGCCACTGACAATC
Ngar_c14400 (*estN1*) _for / _rev	TGTTCAAGACTACGGTGCAGAG	TTATAGTAGCGCCAGCGGATTC
Ngar_c30910 (*estN2*) _for / _rev	CTCTACGGGATAGAAGATATTAAAC	TTGCCATTATAGCAAACACTAC
Ngar_c32780_for / _rev	TAAAGAACGCGATGAAGAATATG	TTCTTGGCACTCATGATATCTC
Ngar_c35080 _for / _rev	AACAAAGAAGCCAAAGGAAGTC	CAAGAGCGTGATAATAGTTACCG
*estN1_Nde*I_for / *_Hind*III_rev	CATATGTATGTCAACGGCTATGC	AAGCTTCTGGTCACTTCTGACCAAG
*estN2_Nde*I_for / *_Hind*III_rev	CATATGCCTTTTCTATCCTCGCCTTC	AAGCTTCCCAACAGGCTTTACATCCTTTGTC
*Ecoli_bioA*_FRT_for /_rev	ATGACAACGGACGATCTTGCCTTTGACCAAC<br>GCCATATCTGGCACCCATAAATTAACCCTCAC<br>TAAAGGGCG	TTATTGGCAAAAAAATGTTTCATCCTGTACC<br>GCGCGGTTAACCGCTGCGGTAATACGACTC<br>ACTATAGGGCTC
*Ecoli_bioB*_FRT_for /_rev	ATGGCTCACCGCCCACGCTGGACATTGTCGC<br>AAGTCACAGAATTATTTGAAATTAACCCTCAC<br>TAAAGGGCG	CCGCGTTGTAATATTCGTCGGTGTCCGGGG<br>TCATCAGCGCCTGTTCAAGATAATACGACT<br>CACTATAGGGCTC
*Ecoli_bioC*_FRT_for /_rev	CAAGCCATTGCAGCGGCATTTGGTCGGGCAG<br>CCGCACACTATGAGCAACAAATTAACCCTCAC<br>TAAAGGGCG	AATCACTCCCAAAAAAAGATGATACGTCAG<br>AGGATATCGCCCCTGCTGTTTAATACGACT<br>CACTATAGGGCTC
*Ecoli_bioD*_FRT_for /_rev	GTTATTTTGTCACCGGAACGGATACCGAAGT<br>GGGGAAAACTGTCGCCAGTAATTAACCCTCA<br>CTAAAGGGCG	CTACAACAAGGCAAGGTTTATGTACTTTCC<br>GGTTGCCGCATTTTCTGGATTAATACGACT<br>CACTATAGGGCTC
*Ecoli_bioF*_FRT_for /_rev	CAGGAGAAAATCAACGCGGCGCTCGATGCG<br>CGGCGTGCTGCCGATGCCCTAATTAACCCTCA<br>CTAAAGGGCG	GCAGACGGTCGATATCCTGCATTTCATGCG<br>CAGCGGTTAGCGTTAAGCGCTAATACGACT<br>CACTATAGGGCTC
*Ecoli_bioH*_FRT_for /_rev	ATGAATAACATCTGGTGGCAGACCAAAGGTC<br>AGGGGAATGTTCATCTTGTAATTAACCCTCAC<br>TAAAGGGCG	CTACACCCTCTGCTTCAACGCCACCAGCAG<br>GTGACAAAACTCGGCCGGATTAATACGACT<br>CACTATAGGGCTC
*Ecoli _bioA_Nde*I_for / *_Hind*III_rev	CATATGACAACGGACGATCTTGCC	AAGCTTTTGGCAAAAAAATGTTTCATCC
*Ecoli _bioB_Nde*I_for / *_Hind*III_rev	CATATGGCTCACCGCCCAC	AAGCTTTAATGCTGCCGCGTTGTAATATTC
*Ecoli _bioC_Nde*I_for / *_Hind*III_rev	CATATGGCAACGGTTAATAAACAAG	AAGCTTCTCACGAGCAATCACTCCC
*Ecoli _bioD_Nde*I_for / *_Hind*III_rev	CATATGAGTAAACGTTATTTTGTCACC	AAGCTTCAACAAGGCAAGGTTTATG
*Ecoli _bioF_Nde*I_for / *_Hind*III_rev	CATATGAGCTGGCAGGAGAAAATCAAC	AAGCTTGTTGCCATGCAGCACCTCC
*Ecoli_bioH_Nde*I_for /*_ Hind*III_rev	CATATGAATAACATCTGGTGGCAGAC	AAGCTTCACCCTCTGCTTCAAC
*Ngar_bioA_Nde*I_for / *_Eco*RI_rev	CATATGAACCTGAGAGATGC	GAATTCGATAGCTTCTCCACTTTTC
*Ngar_bioB_Nde*I_for / *_Hind*III_rev	CATATGATGGCCGATTCAGC	AAGCTTTGCTTTGAGGCCAATCTC
*Ngar_bioD_Nde*I_for / *_Hind*III_rev	CATATGCGCGGCATATTCGTAACAG	AAGCTTCACGGACAGTAGCAGGCTATC
*Ngar_bioF_Nde*I_for / *_Eco*RI_rev	CATATGGCCTCAAAGCATAAGAAG	GAATTCATGATGCCTGTTTTTCTACC
*Ngar_bioC1_Nde*I_for / *_Hind*III_rev<br>(Ngar_c05970)	CATATGTCTGCAGAGGATTCGACTG	AAGCTTTGACGCCGCCCGATAATC
*Ngar_bioC2_Nde*I_for / *_Hind*III_rev<br>(Ngar_c21300)	CATATGCGCTGCCAGTTTACGAAATAC	AAGCTTTGGCTTTTCAGCAGATACTATGG
*Ngar_bioC3_Nde*I_for / *_Hind*III_rev<br>(Ngar_c35810)	CATATGGAAAAGAGGCGTCAACAG	AAGCTTCTTTACGGCCCTGACAAC
*Ngar_bioC4_Nde*I_for / *_Hind*III_rev<br>(Ngar_04400)	CATATGTCCGGTCTTGGAAGCC	AAGCTTGTAAGCGGCGATTATCACCG

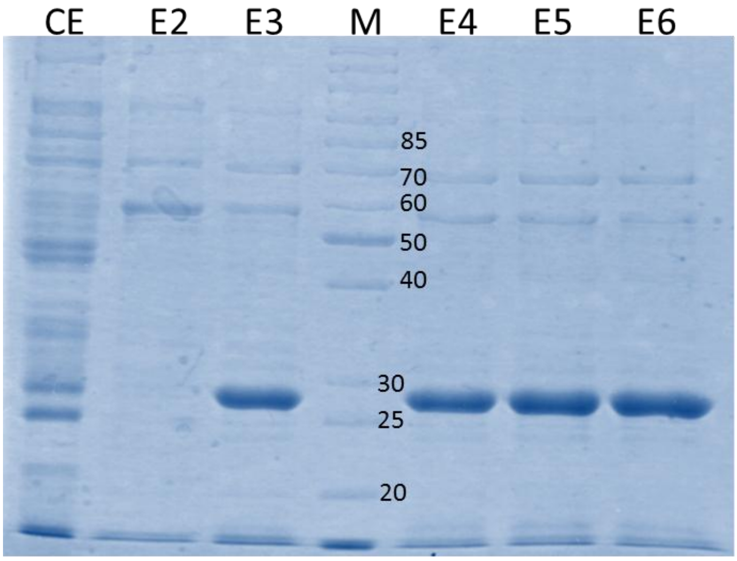

Fig. S1: SDS-PAGE with purified EstN1 visible as strong bands between 25 and 30 kDa. EstN1 was expressed from a pET21a vector in *E. coli* Rosetta-gami 2 (DE3). It was His_6-tag purified via Ni-NTA-agarose. The full-length 12% acrylamide gel shows cell extract (CE) as well as five different elution fractions (E2-E6). M; protein marker (unstained PageRuler #26614, Thermo Scientific) with protein sizes in kDa.

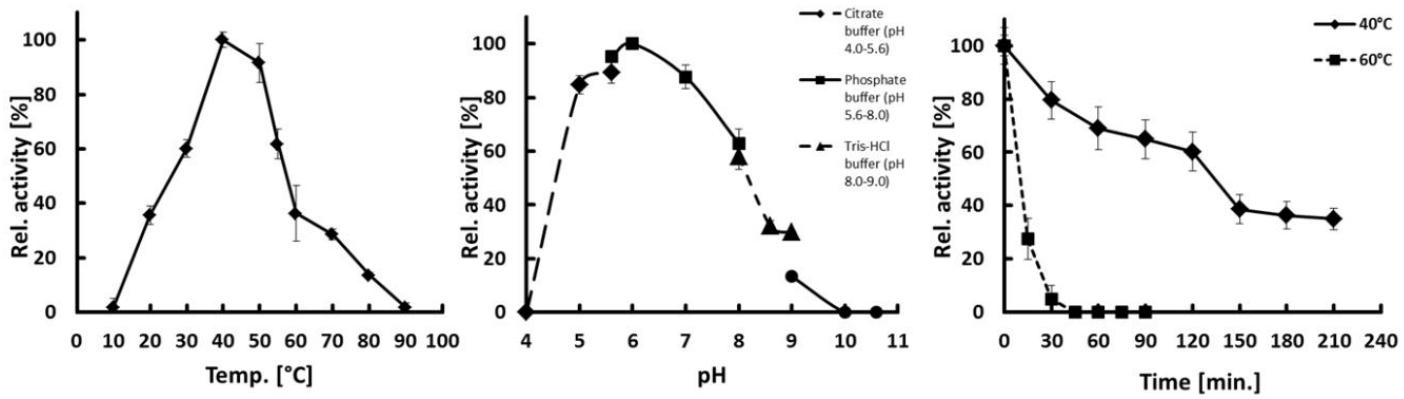

Fig. S2: Biochemical properties of EstN1 tested with *para*-nitrophenol hexanoate as ester substrate measured at E405 nm. EstN1 shows its highest activity at 40°C (left) and is active at a slightly acidic to neutral pH (middle). After 2 hours at 40°C, it still has 60% activity, while incubation at 60°C leads to activity loss after 30 minutes (right).

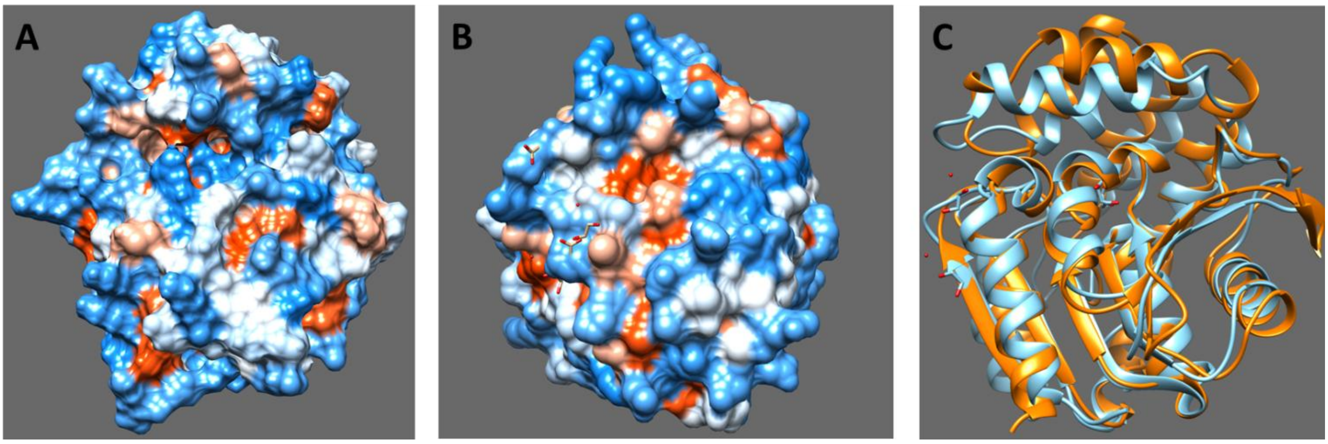

Fig. S3: Surface hydrophobicity of the homology modelled enzyme structure of *N. gargensis* EstN1 (A) and the crystal structure of *E. coli* BioH (B; PDB 1M33). Hydrophobic regions are shown in red. Superimposition of EstN1 (orange) and BioH (blue) is shown in C.

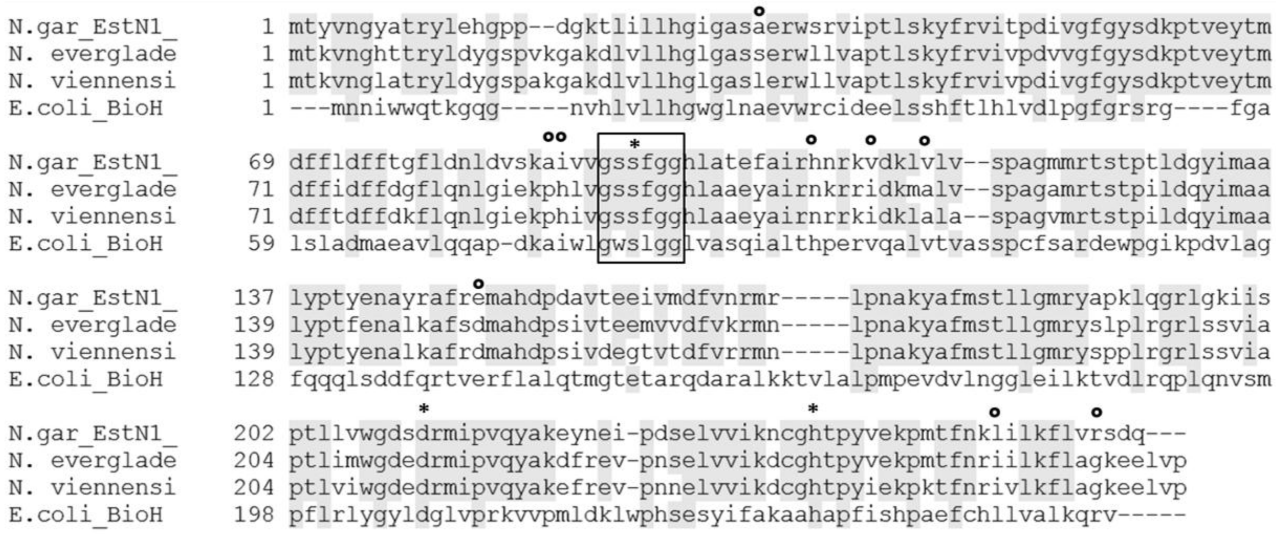

Fig. S4: Global alignment of the amino acid sequences of *N. gargensis* EstN1 and its counterparts from *N. evergladensis* and *N. viennensis* together with *E. coli* BioH. The alignment is based on BLOSUM 62 scoring matrix. While the two archaeal proteins share a 71% identity with EstN1, BioH has only 22% identity to its functional analogue EstN1. The common GXSXGG-motif around the active site serine is framed. Asterisks mark the catalytic triad composed of Ser, His and Asp. Circles mark amino acids that EstN1 and BioH have in common compared to the enzymes from *N. evergladensis* and *N. viennensis*.

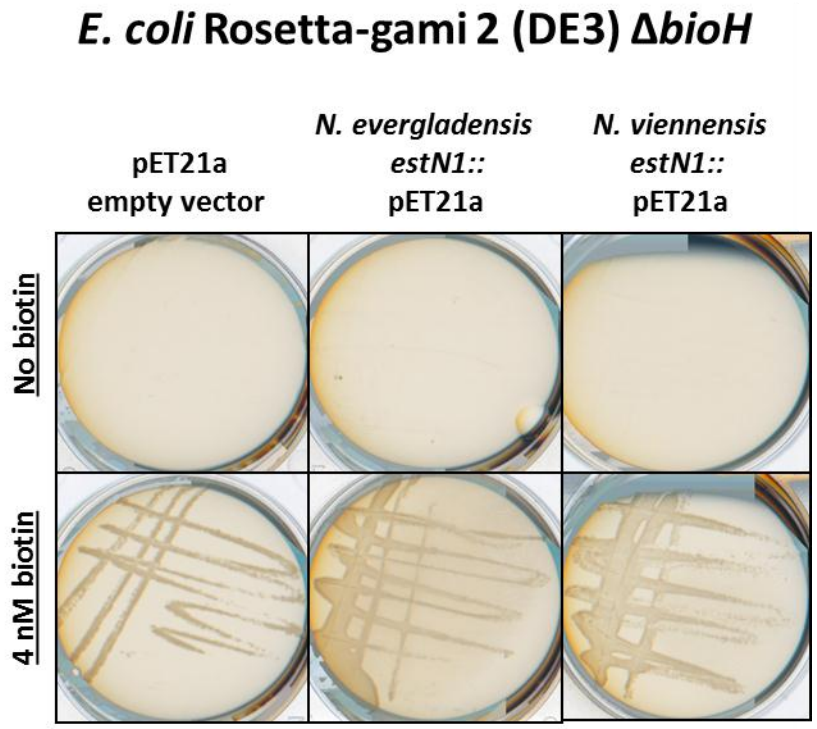

Fig. S5: Complementation of auxotrophic *E. coli* Δ*bioH* insertion mutants with possible *estN1* homologues from *N. evergladensis* and *N. viennensis*. Both enzymes were not able to complement for growth on biotin-free M9 medium.

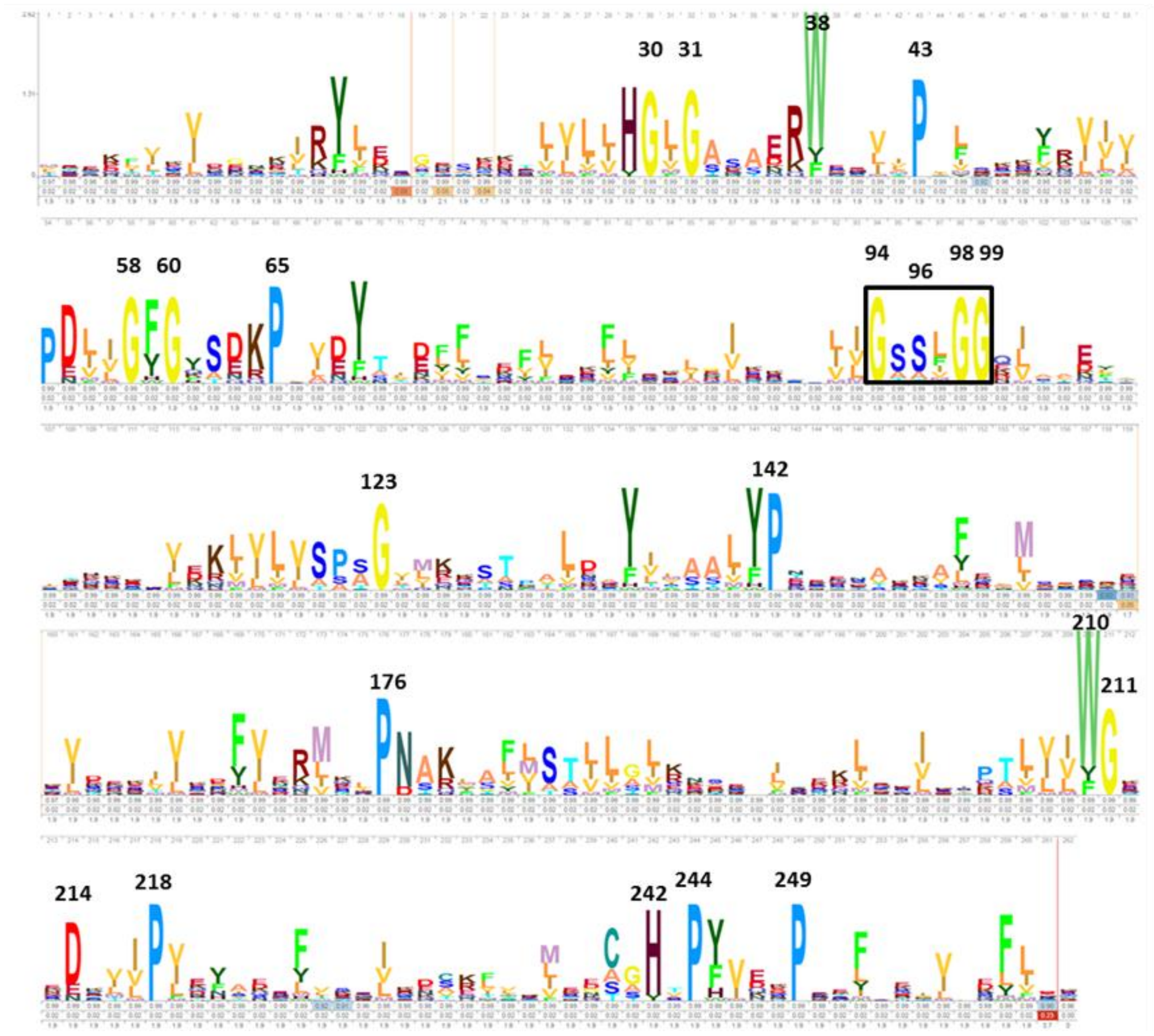

Fig. S6: Hidden-Markov-Model (HMM) logo based on 49 sequences found by Uniprot database searches. Positions of the conserved amino acids are given above the letters. The model was visualized with skylign.org. The conserved GXSXGG motif is framed.
